# “Virgin ureter” vs. “non-virgin ureter”? A comparative analysis on complications and failure of retrograde intrarenal surgery: a multicentre case-control study from RIRSearch Group

**DOI:** 10.1007/s00240-025-01750-z

**Published:** 2025-04-28

**Authors:** Kerem Teke, Naci Burak Çınar, Önder Çınar, Murat Akgül, Cem Başataç, Muhammet Fatih Şimşekoğlu, Hakan Çakır, Oktay Özman, Mustafa Bilal Tuna, Duygu Sıddıkoğlu, Eyüp Burak Sancak, Cenk Murat Yazıcı, Barbaros Başeskioğlu, Haluk Akpınar, Bülent Önal

**Affiliations:** 1 Department of Urology, Kocaeli University School of Medicine, Umuttepe Campus, Kocaeli, 41380 Turkey; 2 Department of Urology, Samsun Medicana Hospital, Samsun, Turkey; 3 Department of Urology, Umraniye Training and Research Hospital, Istanbul, Turkey; 4 Department of Urology, Demiroglu Bilim University, Istanbul, Turkey; 5 Department of Urology, Cerrahpaşa Medical Faculty, Istanbul University, Istanbul, Turkey; 6 Department of Urology, Fulya Acıbadem Hospital, Istanbul, Turkey; 7 Department of Urology, Bahçelievler Memorial Hospital, Istanbul, Turkey; 8 Department of Urology, Acıbadem Maslak Hospital, Istanbul, Turkey; 9 Department of Biostatistics, Çanakkale Onsekiz Mart University, Çanakkale, Turkey; 10 Department of Urology, Çanakkale Onsekiz Mart University, Çanakkale, Turkey; 11 Department of Urology, Namık Kemal University School of Medicine, Tekirdağ, Turkey; 12 Department of Urology, Acibadem Eskisehir Hospital, Eskişehir, Turkey; 13 Florence Nightingale Sisli Hospital, Istanbul, Turkey

**Keywords:** Complication, Flexible ureterorenoscopy, Retrograde intrarenal surgery, Ureteral virginity, Virgin ureter

## Abstract

It is unclear whether ureteral virginity has an effect on retrograde intrarenal surgery (RIRS). We aimed to evaluate the impact of ureteral virginity on RIRS outcomes in a multicenter study. Data from the RIRSearch study group database were retrospectively reviewed. Patients with a history of endoluminal interventions or extrinsic ureteral surgery were categorized as having a “non-virgin ureter,” while those without such histories were classified as “virgin ureters.” Case-control matching was performed based on age, gender, uretral access sheath size, and stone characteristics. Demographic, clinical, surgical and complication data were compared after-matching. A total of 894 procedures were included, with 119 (13.3%) involving non-virgin ureters. Pre-matching, the non-virgin ureter group had higher mean age (50.6 ± 13.2 vs. 46.6 ± 13.6 years) and Charlson comorbidity index ≥ 2 (51.3% vs. 40.4%). In addition, number of stones, total-stone volume and rate of multiple stone localization were significantly higher in non-virgin ureter group. Operation time, hospital stay, surgical failure, need for auxiliary treatment, and perioperative complications were significantly higher in non-virgin ureter group (*p* < 0.05). After case-matching, perioperative complications (18.7% vs. 5.3%), hospital stay (1.54 ± 1.30 vs. 1.18 ± 0.98 days), and auxiliary treatment requirements (20% vs. 8.4%) remained significantly higher in non-virgin ureter group (*p* < 0.05). There was no significant difference in postoperative complication rates (17.3% vs. 19.8%) or surgical failure rates (36% vs. 26%). Non-virgin ureters were associated with higher perioperative complication rate, longer hospital stays and increased need for auxiliary treatments during RIRS. Patients with non-virgin ureters may be informed about these potential risks before surgery.

## Introduction

Retrograde intrarenal surgery (RIRS) is the gold standard treatment method for upper urinary tract stones smaller than 2 cm. The procedure involves first passing through the urethra and bladder to reach the upper urinary tract, specifically the ureter and renal collecting system. Laser lithotripsy is then performed to treat the stones. There are many factors affecting surgical success/failure in RIRS, and complications that may occur during and after surgery. Generally, these are excess stone size, stone density, lower pole stones and whether or not ureteral stents are present before surgery [1, 2].

There are reports indicating that previous ureteral interventions have altered the anatomical or functional properties of the ureter [3, 4]. However, there does not appear to be any study investigating RIRS outcomes between patients without a history of ureteral intervention and those with a history of ureteral intervention. The ureter without history of extrinsic or intrinsic intervention may be defined as virgin, and we hypothesized that RIRS outcomes may be different between patients with virgin ureter and those with non-virgin ureter. The aim of this study was to evaluate the impact of ureteral “virginity” status on RIRS failure and complication rates through a multicenter retrospective study using a case-controlled approach.

## Materials and methods

### Patients and data collection

Consecutive RIRS procedures between 2010 and 2023 were retrospectively reviewed from the RIRSearch study group database. Patients with ureteral stents prior to RIRS, patients younger than 18 years of age, patients who had a history of pelvic radiotherapy or non-ureteral pelvic surgery, and patients in whom there was no attempt to insert a ureteral access sheath (UAS) during RIRS were excluded from the study. Retrieved demographic variables were age, gender, body mass index (BMI), age-adjusted Charlson Comorbidity index (CCI), and history of previous ipsilateral ureteral intervention. Clinical data collected included side of surgery, serum creatinine level, stone density in Hounsfield units (HU), stone burden, presence of hydronephrosis, stone number and stone location. Surgical and complication data were the requirement for active ureteral dilatation (coaxial or balloon dilatators or optical dilatation using ureteroscope), the UAS size used in both successful and unsuccessful placements, JJ stent insertion at the end of the RIRS, the type of flexible ureteroscope employed, the fluoroscopy time, the hospitalization time and perioperative and early postoperative (˂30 days) complications. Perioperative and postoperative complications were evaluated according to the Modified Satava Classification (MSC) and Modified Clavien Classification (MCC) systems, respectively. Finally, surgical success or failure data were obtained from the follow-ups after RIRS procedures. Surgical failure criterion was defined as a residual stone of 4 mm or more in non-contrast computed tomography (CT) taken within 1–3 months of the RIRS procedure [5–10]. To access the upper urinary tract any additional ipsilateral intervention session after RIRS was denoted as an auxiliary procedure.

Patients included in the study who did not have a history of ipsilateral ureteral intervention were categorized as “virgin ureter”. Patients who had a history of any endoluminal/intrinsic intervention (history of antegrade or retrograde stent placement in any urological procedure such as percutaneous nephrolithotomy or pyelolitotomy, and/or history of ureteroscopy) or extrinsic surgery (ureterolithotomy, ureteroneocystostomy) for the ipsilateral ureter were categorized as “non-virgin ureter”. Those two groups were compared in terms of demographic, clinical, and RIRS outcome (surgical success and complications) before and after the case-control matching approach. In order to further evaluate the renal collecting system complexity of patients requiring second RIRS session due to residual stones, the infundibulopelvic angle (IPA) was also measured with non-contrast CT in non-virgin ureter group, as previously described [11]. This study was conducted after approval by the ethics committee of Kocaeli University School of Medicine (Approval No.: GOKAEK-2025/02/01).

### Case-control matching

To reduce the risk of bias and potential confounding factors, we adopted a case-control study design procedure of Statistical Package for the Social Sciences (SPSS) version 19.0 software (IBM Corp., Armonk, NY, USA). The non-virgin group was individually matched at a ratio of approximately 1:2 to patients with virgin ureter using an optimal matching approach based on confounders, including age (within 4 years), gender, UAS size used or attempted (≤ 10–12 Fr and 11–13 Fr≤), stone location, stone volüme (within 300 mm^3^), stone density, and stone number. After adjusting for confounders, the groups were compared in terms of demographic, clinical, surgical, complication, and success data.

### Surgical procedure

All patients undergoing RIRS had a negative urine culture prior to the procedure. Non-contrast CT was used for the diagnosis of patients before RIRS. Consent forms were obtained from all patients prior to RIRS. RIRS procedures were performed under general anesthesia and in the lithotomy position. The surgical procedure was preceeded by cystourethroscopic examination, the insertion of a Sensor™ guide (Boston Scientific, USA) into the ureter, the demonstration of upper tract anatomy using retrograde pyelograpy, and the placement of a UAS over the sensor guidewire. The UASs employed in this study were Flexor^®^ (Cook Urological, Spencer, IN, USA), Bi-FlexTM (Rocamed, Monaco), and Navigator^®^ HD (Boston Scientific, Marlborough, MA, USA). Ureteral dilatation was performed using balloon (UroMax UltraTM, Boston Scientific), coaxial dilator (8–10 F, Boston Scientific) or optical dilatation with ureteroscope (8/9.8 F ureteroscope, Richard Wolf medical instruments Knittlingen, Germany; or 8–9 F, Karl Storz, Rietheim-Weilheim, Germany) before or after UAS placement, based on surgeon’s preference. Detailed ureteral dilatation approaches have been described in previously reported studies by the RIRSearch group [8, 9, 10, 12]. Despite lower ureteral dilatation if UAS could not be inserted, a flexible ureteroscope (fURS) was inserted into the upper urinary tract with a backloading approach over the guidewire. The fURSs used in RIRS procedures were Karl Storz Flex-X2 (Karl Storz, Tuttlingen, Germany), WiScope^®^ (OTU Medical Inc.), and Uscope UE3022 (Pusen™). A Ho: YAG laser was used for laser lithotripsy. Energy pulse frequency and power were adjusted according to stone hardness and burden. Stones larger than 2–3 mm were removed from the body with a nitinol-coated basket [8, 9, 10, 12].

### Statistics

Categorical variables are expressed as count/frequency (n) and percentage (%). To assess the normality hypothesis for the continuous variables, the Shapiro–Wilk test was used. Continuous variables are presented as mean ± standard deviation (SD) or median and interquartile range (IQR); analyzed with independent samples t test or Mann Whitney U test as appropriate. Categorical variables were evaluated using the Chi-square test and Fisher exact test. Univariate analysis by binary logistic regression analysis was performed to evaluate whether the risk of complications and auxiliary interventions in the non-virgin ureter group compared to the virgin ureter group after case-control matching. All statistical analyses were conducted using SPSS version 19.0 software (IBM Corp., Armonk, NY, USA). A *p* < 0.05 was considered statistically significant.

### Results

**Fig. 1:**
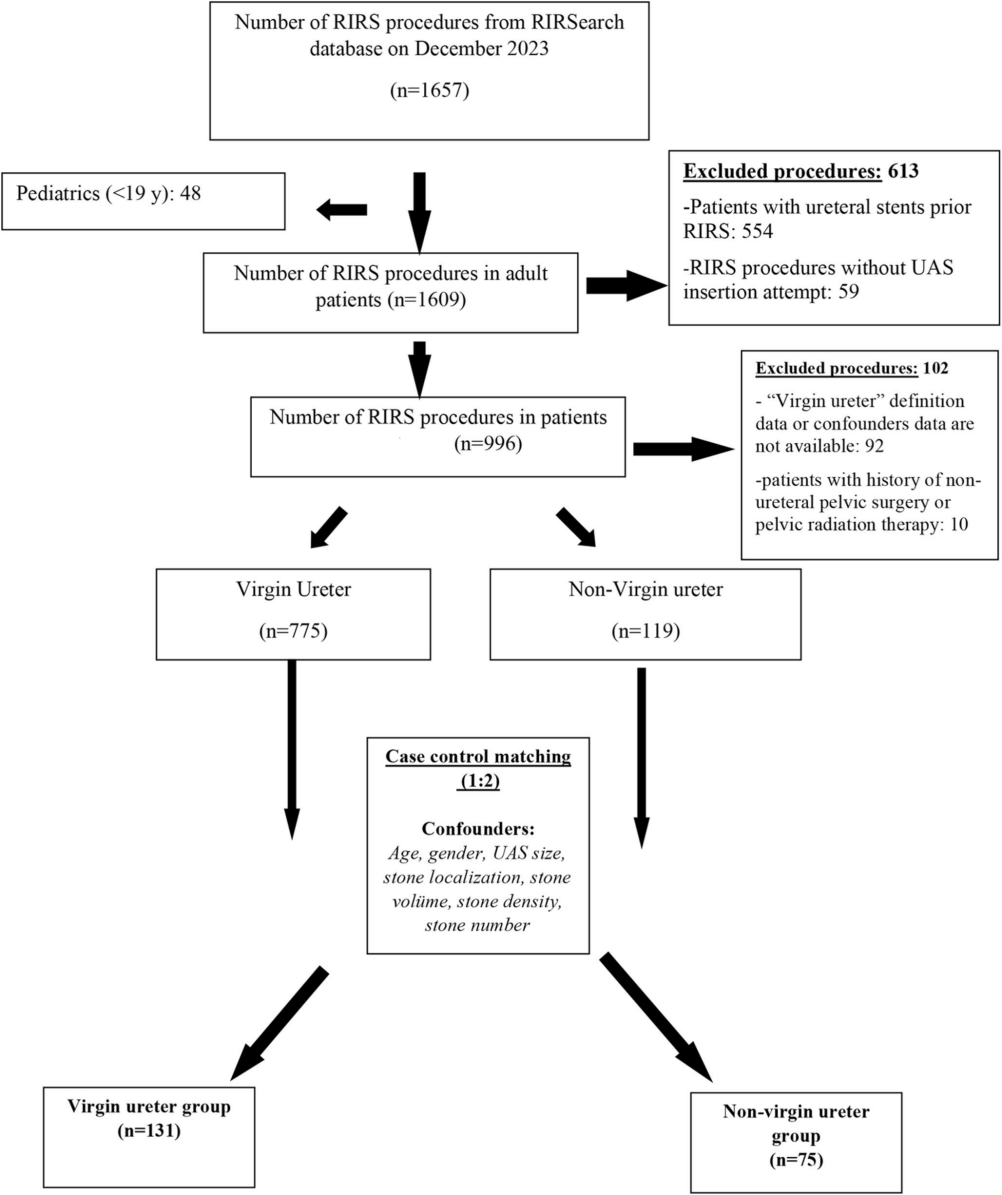
Flowchart of the study population selection and case control matching for the virgin and non-virgin groups

A total of 894 procedures in 819 patients that met the inclusion criteria were included in the study. The mean age of patients was 47.2 ± 13.6 years, the proportion of male patients was 61.6%, and the median CCI was 1 (0–1). RIRS procedures were performed by five different surgeons (CMY: 619, HC: 187, KT: 36, MFŞ: 17, EBS: 35). The “non-virgin ureter” group consisted of a total of 119 (13.3%) procedures (Fig. 1). Of the patients in the “non-virgin ureter” group, 84 (70.6%) had a history of only one ureteroscopy, 25 (21%) had two or more ureteroscopies, 10 (8.4%) had a history of antegrade or retrograde ureteral stenting (2 in percutaneous nephrolithotomy procedures, 3 in pyelolithotomy procedures, and 5 in retrograde DJ stenting for obstruction). There was no patients with extrinsic surgery for ipsilateral ureter based on study protocol. There was no case of failed ureteroscopy due to uncompliant or strict ureters among virgin group and non-virgin ureter group.

Before case-control matching, mean age (50.6 ± 13.2 vs 46.6 ± 13.6 years) and the rate of patients with CCI > 1 (51.3% vs 40.4%) were significantly greater in the “non-virgin ureter” group (*p* < 0.05). In the “non-virgin ureter” group, the multiple stone rate (56.3% vs 35.6%), total stone volume (895.2 (284.3-2441.3) vs 503 (205.5-1049.8) mm^3^) and multifocal stone location rate (54.6% vs 30.7%) were significantly higher compared to the “virgin-ureter” group (*p* < 0.05). Moreover, the operation time (81.7 ± 36.2 vs 65.8 ± 35.6 min), hospital stay (1.74 ± 1.75 vs 1.50 ± 1.72 days), perioperative complication rates (17.6 vs 6.5%), auxiliary treatment requirement (31.1 vs 8%), and surgical failure rate (47.9% vs 20.8%) were significantly greater in the “non-virgin ureter” group (p < 0.05). There was no further statistical difference between the groups in terms of demographics, clinical, surgical, and complication data.

A hundred and thirty-one patients from the “virgin ureter” group and 75 patients from the “non-virgin ureter” group (1:2) were matched using a case-control method in terms of age, gender, total stone volume, stone location, stone density, number of stones and similar UAS use (≤ 10–12 Fr and 11–13 Fr≤). Of the 75 with non-virgin ureter, 53 (70.7%) had one and 17 (22.7%) had more than one ureteroscopy, whereas 5 (6.7%) had only undergone ureteral stenting. After matching, apart from demographic and clinic data, the UAS usage, and the UAS insertion failure rate, the lower ureteral dilatation rate, the fluoroscopy time, and the surgical time were also similar between the virgin and non-virgin ureter groups (*p* > 0.05). Moreover, there was no significant difference for the postoperative complication rates (17.3% for non-virgin ureter group vs. 19.8% for virgin ureter group) and the surgical failure rates (36% for non-virgin ureter group vs. 26% for virgin ureter group) (*p* > 0.05). However, when the perioperative complication rate (18.7% vs. 5.3%), hospital stay (1.54 ± 1.30 vs. 1.18 ± 0.98 days) and auxiliary treatment requirement rate (20% vs. 8.4%) were compared, these were significantly worse in the “non-virgin ureter” group compared to the “virgin ureter” group (Table 1).

**Table 1:** Comparison of demographic, clinical, operative and postoperative data of patients in the “non-virgin ureter” and “virgin ureter” groups who underwent retrograde intrarenal surgery before and after case control matching

	*Before case control matching (* *n = 894)*			*After case control matching (* *n = 206)*		
	Virgin ureter	Non-virgin ureter	*P*	Virgin ureter	Non-virgin ureter	*P*
Number of procedures, n (%)	775	119		131	75	
Age (years), (mean ± SD)	46.6 ± 13.6	50.6 ± 13.2	0.003*	47.2 ± 12.6	49.3 ± 13.1	0.26*
Gender, n (%)						
Male	478 (61.7)	67 (56.3)	0.268^**µ**^	78 (59.5)	44 (58.7)	0.902 ^**µ**^
Female	297 (38.3)	52 (43.7)		53 (40.5)	31 (41.3)	
BMI (kg/m^2^) (mean ± SD)	27.4 ± 4.2	27.4 ± 4.6	0.928*	27.8 ± 4.7	27.3 ± 4.9	0.58*
Age-adjusted CCI, n (%)						
0–1	462 (59.6)	58 (48.7)	**0.025** ^**µ**^	71 (54.2)	43 (57.3)	0.663 ^**µ**^
≥ 2	313 (40.4)	61 (51.3)		60 (45.8)	32 (42.7)	
Presence of preoperative hydronephrosis, n (%)	336 (43.4)	46 (38.7)	0.251 ^**µ**^	59 (45)	28 (37.3)	0.281 ^**µ**^
Preoperative creatinine, (mean ± SD)	0.96 ± 0.51	0.83 ± 0.16	0.211*	0.94 ± 0.14	0.81 ± 0.17	0.121*
Postoperative creatinine, (mean ± SD)	0.89 ± 0.27	0.96 ± 0.33	0.107*	0.87 ± 0.23	0.93 ± 0.28	0.404*
Number of stones, n (%)						
Single	490 (63.2)	52 (43.7)	**0.01** ^**µ**^	62 (47.3)	35 (46.7)	0.927 ^**µ**^
Multiple	276 (35.6)	67 (56.3)		69 (52.7)	40 (53.3)	
Side, n (%)			0.808 ^**µ**^			0.087 ^**µ**^
Right	393 (50.7)	59 (49.6)		79 (60.3)	36 (48)	
Left	381 (49.2)	60 (50.4)		52 (39.7)	39 (52)	
Total stone volume, mm^3^ (median, IQR)	503 (205.5-1049.8)	895.2 (284.3-2441.3)	**0.002** ^**¶**^	641.8 (285.1–1248)	690 (323.2-1383.3)	0.782^**¶**^
Stone density value (HU) (mean ± SD)	996.8 ± 302.7	1028.1 ± 290.7	0.295*	1008.9 ± 278.6	992.6 ± 247.1	0.67*
Stone location, n (%)						
Upper Pole	23 (3)	0	**0.01** ^**µ**^	0	0	0.980 ^**µ**^
Middle Pole	41 (5.3)	5 (4.2)		5 (3.8)	3 (4)	
Lower Pole	96 (12.4)	8 (6.7)		11 (8.4)	6 (8)	
Renal Pelvis	201 (25.9)	25 (21)		25 (19.1)	15 (20)	
Upper Ureter	175 (22.6)	16 (13.4)		22 (16.8)	12 (16)	
Multiple locations	238 (30.7)	65 (54.6)		68 (51.9)	39 (52)	
Size of UAS, n (%)			0.23 ^**µ**^			0.960 ^**µ**^
none	120 (15.5)	12 (10.1)		12 (9.2)	6 (8)	
9.5–11.5 Fr	30 (3.9)	19 (16)		4 (3.1)	16 (21.3)	
10–12 Fr	430 (55.5)	62 (52.1)		86 (65.6)	36 (48)	
11–13 Fr	67 (8.6)	15 (12.6)		8 (6.1)	8 (10.7)	
12–14 Fr	117 (15.1)	10 (8.4)		21 (16)	9 (12)	
Lower ureteral dilatation, n (%)	510 (65.8)	85 (71.4)	0.226 ^**µ**^	100 (76.3)	55 (73.3)	0.631 ^**µ**^
Fluoroscopy time, (mean ± SD)	54.8 ± 105	58.8 ± 149	0.642*	51.6 ± 97	86.5 ± 180.3	0.404*
Surgical time, min, (mean ± SD)	65.8 ± 35.6	81.7 ± 36.2	0.0001*	66.2 ± 28.5	74.3 ± 31.7	0.105*
Length of hospital stay, (days) (mean ± SD)	1.50 ± 1.72	1.74 ± 1.75	0.007*	1.18 ± 0.98	1.54 ± 1.30	0.002*
Perioperative complications, n (%)	50 (6.5)	21 (17.6)	**0.0001** ^**µ**^	7 (5.3)	14 (18.7)	**0.002** ^**µ**^
Postoperative complications, n (%)	152 (19.6)	24 (20.2)	0.892 ^**µ**^	26 (19.8)	13 (17.3)	0.70^**µ**^
Surgical failure, n (%)	161 (20.8)	57 (47.9)	**0.0001** ^**µ**^	34 (26)	27 (36)	0.093^**µ**^
Need for secondary treatment, n (%)	62 (8)	37 (31.1)	**0.0001** ^**µ**^	11 (8.4)	15 (20)	**0.012** ^**µ**^

Abbreviations: BMI; Body Mass Index. CCI; Charlson comorbidity index. UAS; Ureteral access sheath. HU; Hounsfield Unit. SD; Standard Deviation. IQR;Inter Quantile Range **¶**: Mann Whitney U, *: Independent samples t Test, **µ**: Chi-Square Test

The details of the perioperative and the postoperative complications in non-virgin and virgin ureter groups undergoing RIRS are presented in Table 2. All of the perioperative complications in the non-virgin ureter group were an inability to reach the stone that was graded by MSC as grade 2b (14 of 75, 18.7%). All of these 14 patients were considered to have failed RIRS procedures due to residual stones that could not be reached. This complication rate was significantly lower in the virgin ureter group compared to non-virgin ureter group (4.6% vs. 18.7%, *p* < 0.05. There were 6 patients in the virgin ureter group who underwent the second session RIRS procedure as an auxiliary treatment because the stone could not be reached. The elapsed time to the auxiliary treatment from first RIRS procedure was 132.8 ± 216.5 days and the surgical success rate in these 6 patients was 83.3%. Moreover, 13 of these patients in non-virgin ureter group who had failure of initial RIRS and accepted auxillary treatment underwent a second RIRS session after a mean of 51.7 ± 23.2 days, and two patients from the non-virgin ureter group who had stent migration underwent stent removal using ureteroscopy under general anesthesia. Thus, the rate of auxiliary procedure (a second endoscopic session) was significantly lower in the virgin ureter compared to the non-virgin ureter group (4.6% vs. 17.3%, *p* < 0.05, Table 3). It was noteworthy that the stone localization of 13 patients who underwent second RIRS session was predominantly in the lower calyx and surgical success was achieved in only 6 of these procedures (46.2%). Of 13 patients, 6 was male and median age was 53 (44–59). In order to assess the complexity of lower caliceal anatomy, the IPA values were also calculated in non-virgin ureter group and median IPA level of these 13 procedures was 32 (29–39). Minor (Grades 1 and 2) and major (Grades 3-4b) postoperative complications (MCC system) were similar between the patients with virgin and non-virgin ureter (*p* > 0.05).

**Table 2:** The classifications of perioperative and postoperative complications by the modified SATAVA and the modified clavien classifications systems in patients with Virgin ureter and non-virgin ureter who underwent retrograde intrarenal surgery

*After case control matching*, *n* = 206			Virgin ureter *n* = 131	Non-virgin ureter *n* = 75	*p*
**Modified Satava complications system**					
	1	Bleeding	1 (0.8)	-	**0.002**
	2b	Inability to reach the stone in the lower pole or residual stone	6 (4.6)	14 (18.7)	
Total			7 (5.3)	14 (18.7)	
***Modified Clavien classification system***					
***Minor***	1	Renal colic	15 (11.5)	5 (6.7)	0.430
	2	Fever requiring antibiotics	6 (4.6)	4 (5.3)	
***Major***	3b	JJ stent migration requiring correction	4 (3.1)	3 (4)	0.608
	3b	Steinstrasse	1 (0.7)	-	
	4b	Urosepsis	-	1 (1.3)	
Total			26 (19.8)	13 (17.3)	

**Table 3:** Auxiliary surgical treatments following case controlling

*n* = 206			*p*
	Virgin ureter (*n* = 131)	Non-virgin ureter (*n* = 75)	
Flexible URS	6 (4.6)	13 (17.3)	
Semirigid URS	1 (0.8)	-	
Pigtail catheter removal with general anesthesia	2 (1.5)	2 (2.7)	
PCNL	2 (1.5)	-	
Total	11 (8.4)	15 (20)	0.012

Abbreviations: PCNL; Percutaneous nephrolithotomy, URS; Ureteroscopy

**Fig. 2:**
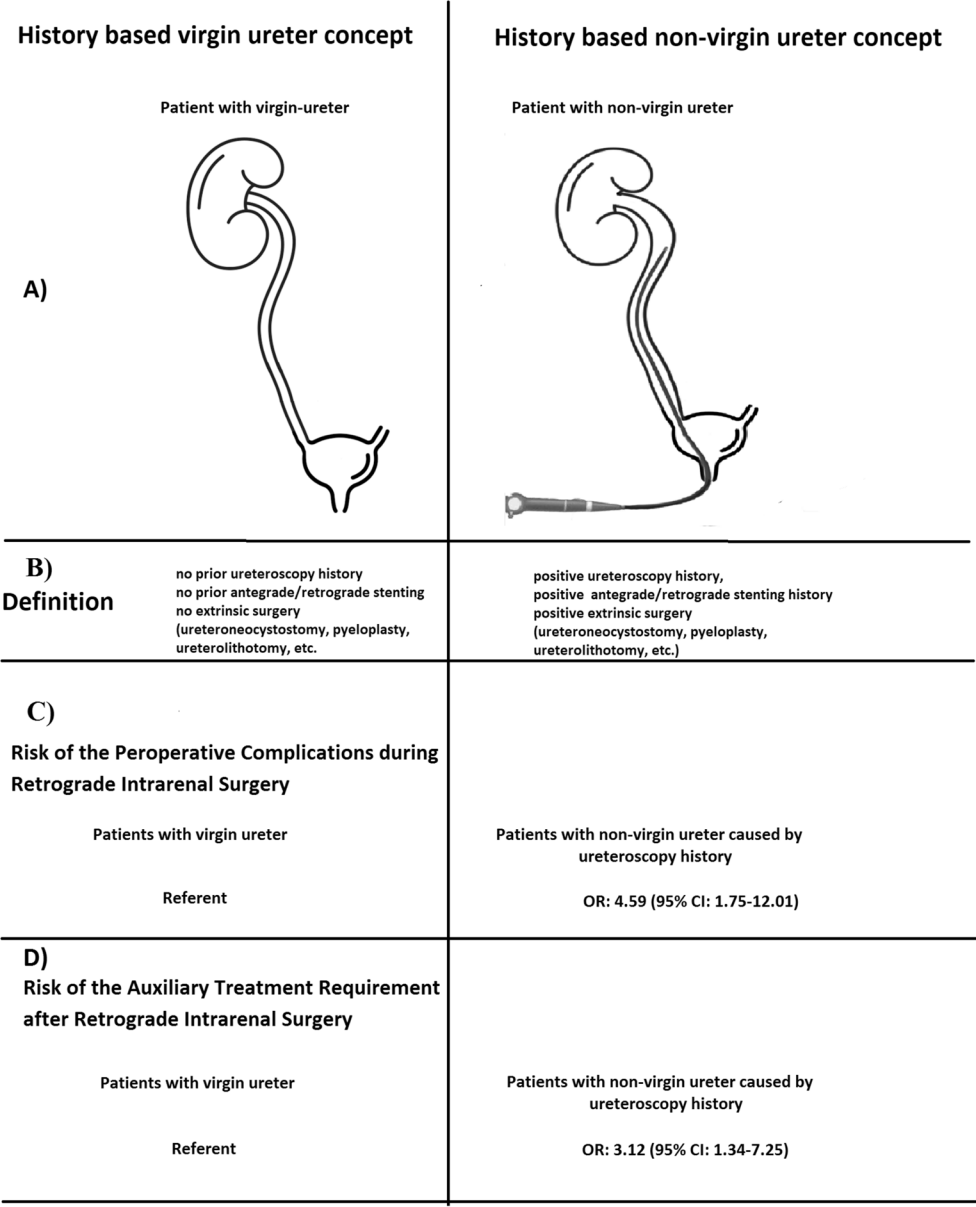
Schematic draw the virgin- and non-virgin ureteral concept (**A**). Intrinsic and extrinsic factors that can be associated the loss of ureteral virginity (**B**). Assessment the risk of Peroperative complications during Retrograde Intrarenal Surgery between patients with virgin ureter and patients with non-virgin ureter (**C**). Assessment the risk of auxiliary treatment requirement after Retrograde Intrarenal Surgery between patients with virgin ureter and patients with non-virgin ureter (**D**)

After case-control matching (*n* = 206), the perioperative complication risk and the auxiliary treatment requirement risk were compared in the non-virgin ureter group and the virgin-ureter group in terms of detail of the history of ureteral intervention. The ureteroscopy history (*n* = 70), one of the endoluminal/intrinsic etiologies of non-virgin ureter gave rise to a significant increase in the perioperative complication risk (OR: 4.59, 95% CI: 1.75–12.01) and the auxiliary intervention requirement risk (OR: 3.12, 95% CI: 1.34–7.25) compared to virgin ureter (*n* = 131) (Fig. 2). Antegrade or retrograde ureteral stenting (*n* = 5) did not increase the perioperative complication risk or the auxiliary procedure requirement risk (*p* > 0.05).

## Discussion

In this study, patients with a history of “endoluminal or external”, that is intrinsic or extrinsic interventions in the ipsilateral ureter were classified as having a ‘non-virgin ureter,’ while the absence of such history was defined as having a ‘virgin ureter.’ After clearly defining the ureteral status, we retrospectively evaluated the impact of a non-virgin ureter caused by intrinsic etiologies on RIRS outcomes, specifically assessing surgical success rates, complication rates, and the need for additional treatments in a multicentre case-control study. To the best of our knowledge, this is the first study to examine the effect of ureteral virginity on RIRS outcomes. While overall surgical success and postoperative complication rates were not different between the groups, perioperative complications and the need for auxiliary treatments were significantly higher in the non-virgin ureter group. Notably, patients with a history of prior ureteroscopy had an approximately 4.5-fold increase in perioperative complication risk based on “inability to reach stone” and nearly a 3-fold increase in the need for auxiliary treatments (predominantly a second RIRS session) compared to those with virgin ureters, which is a finding, we believe, that warrants attention.

Regarding the terminology of virgin ureters, various studies have defined ‘virgin ureters’ differently and incompletely. One study used this term to describe stentless patients to examine the relationship between the use of UAS in stented ureters and postoperative ureteral strictures [13]. In another study, patients without prior UAS placement were defined as “virgin ureters” to assess the failure rates of UAS placement during primary fURS [14]. In another study investigating the predictive factors for UAS placement success and postoperative outcomes, primary fURS cases were defined as virgin ureters [15]. In a recent study examining the effect of preoperative double J stenting on surgical outcomes, patients who did not receive a stent were described as having a virgin ureter [16]. In another recent study examining the effect of distal ureteral lateralization angle on successful UAS placement, the definition of virgin ureter was used for patients with primary urolithiasis who had not previously undergone ureteroscopy for any reason, had not received ureteral stent placement, and had not received extracorporeal shock wave lithotripsy (ESWL) [17]. The definition used in our study is based on extrinsic or intrinsic interventional history to offer a broader and more precise characterization than those in previous studies. In other words, we assumed that any factor that has an anatomical or functional effect on the ureter would disrupt the virginity and, therefore, nature of the ureters in terms of surgical outcome. However, according to our findings, it seems that the loss of virginity caused by intrinsic etiology, such as ureteroscopy in our cohort, did not present an obstacle in passing through the ureter but, in contrast, was associated with an inability to reach stone due to some factor associated with lower calyceal anatomy.

Previous ureteral surgeries can lead to undesired anatomical changes, and it has been shown that stent placement may induce functional alterations by impacting ureteral peristalsis [3, 18]. The incidence of ureteral strictures following ureteroscopy for ureteral stones ranges from 0.1 to 5.8% [19, 20], with stone impaction identified as a major risk factor for postoperative ureteral stricture [4, 21]. In the present study, in RIRS procedures performed for patients with non-virgin ureters due to intrinsic etiologies, the rate of UAS employed, and the UAS insertion failure rate, the lower ureteral dilatation rate, the fluoroscopy time, and the surgical time did not differ from virgin ureter patients. That is, a history of previous endoluminal intervention may not constitute a significant anatomical or functional deterioration to accessing the renal collecting system for patients who are candidates for RIRS. We speculate that the perioperative complication of “inability to reach the stone” is not likely attributable to the anatomical and functional changes associated with non-virgin ureters.

We highlighted that all of the perioperative complications were inability to reach the stone in patients with non-virgin ureters and subsequent requirement of a second RIRS session as an auxiliary procedure. We argue that possible reasons for this may be failure to reach the stone due to congenital or acquired complex calyceal anatomy (narrow infundibulopelvic angle, narrow calyceal infundibulum). Acquired lower calyceal anatomic alteration may be due to inflammatory changes at this level in patients with a history of ureteroscopic intervention. This may be related to the inability to reach the stone for patients with non-virgin ureters in RIRS procedure. Another hypothetical mechanism is that large fragments that are inadequately fragmented during RIRS dislocate to the lower calyces and, therefore, may lead to the inability to reach the stone, especially in non-virgin ureter patients with difficult congenital lower calyceal anatomy. Moreover, non-opaque stones may not be defined in a challenging RIRS session, even if fluoroscopy is used. This would increase the need for second procedures. We believe that preoperative identification of caliceal anatomic properties in cases with non-virgin ureters may reduce the need for a second auxiliary procedure in these patients. Therefore, in patients with non-virgin ureters, the high rates of failure to access the stone make the use of fluoroscopic evaluation during RIRS procedures and detailed preoperative radiographic examination essential. We consider that both effective intraoperative fluoroscopy use and preoperative urographic diagnostic evaluation, such as CT urography or intravenous pyelography may be a good strategy, rather than non-contrast CT imaging only for non-virgin ureter patients who are candidates for a RIRS procedure. These imaging methods may be effective in achieving higher stone-free rates and thus reducing the need for auxiliary procedures, as they will facilitate the evaluation of important risk factors, such as narrow IPA [22]. Recently, a systematic review and meta-analysis has shown that the stone-free rate is high as the IPA value increases above 50^o^, while the stone-free rate decreases with lower IPA value and especially it has been shown that no patient achieves stone-free status below 30^o^ [23]. Given the median IPA level was 32 in the non-virgin ureter patients who underwent second session RIRS, evaluating the complexity of the lower calyceal anatomy (IPA, etc.) before treating a lower calyceal stone in a patient with non-virgin ureter seems essential for preoperative counseling.

Cases in which stents were placed and postponed to the next session due to uncompliant or strict ureters can be encountered in the literature [24]. However, the absence of such cases among the patients included in the study protocole can be explained by the fact that the rate of active lower ureteral dilatation, which reached a rate of 76% in our study, was particularly high compared to the literature [24].

The first limitation of this study is its retrospective design. However, this limitation has been addressed by conducting a multi-center, case-controlled study, enhancing the reliability of the results. Second, although we included extrinsic ureter surgeries in the definition of non-virgin ureter, there were no patients with extrinsic intervention in our study (all had intrinsic pathology). Further studies are needed to elucidate the results of RIRS in patients with non-virgin ureter caused by extrinsic interventions. Third, our study includes the data of RIRS procedures performed only under general anesthesia. However, we acknowledge that RIRS procedures can be also performed under spinal anesthesia [25].

## Conclusions

Our observational multicentre study indicated that perioperative complications, hospitalization duration, and the need for auxiliary treatments were significantly higher in RIRS procedures performed on patients with non-virgin ureters compared to those with virgin ureters, based on our methodological definition of virgin ureter. Therefore, we propose that patients with non-virgin ureters caused by intrinsic interventions may be informed prior to RIRS about the potential perioperative complications, such as inability to reach some stones. For patients with non-virgin ureters, both preoperative urographic diagnostic tools prior to surgery and effective fluoroscopic examination during RIRS may aid in identification of the renal calyceal anatomy and therefore reduce the rate of the “inability to reach stone” complication and thus the need for auxiliary treatment like a second RIRS session. Consequently, to avoid auxiliary procedures, other minimally invasive treatment methods, such as mini percutaneous nephrolithotomy and endoscopic combined intrarenal surgery, instead of RIRS, may be considered in non-virgin ureter cases with complex lower calyceal anatomy.

Take home messages of this study are: the ureter with a history of intrinsic or extrinsic interventions should be defined as “non-virgin”; according to our findings, patients with non-virgin ureter who are candidate to undergo ipsilateral RIRS are likely to be at high-risk for perioperative complications, such as “inability to reach stone”, and for needing the auxiliary intervention; in our study, we found and highlighted that one of the descriptive features belonging to cases with non-virgin ureter was also the narrow infundibulopelvic angle; therefore, if RIRS is planned for cases with non-virgin ureters, we propose that the lower caliceal anatomy should be evaluated prior to surgery and that other minimally invasive treatment options, instead of RIRS, may be kept in mind for better stone-free rates in cases with narrow infundibulopelvic angle.

## Data Availability

No datasets were generated or analysed during the current study.
